# Investigation of Signal Transmission Dynamics in Rulkov Neuronal Networks with Q-Learned Pathways

**DOI:** 10.3390/e27080884

**Published:** 2025-08-21

**Authors:** Mio Kobayashi

**Affiliations:** Institute of Humanities, Shinshu University, 3-1-1 Asahi, Matsumoto 390-8621, Nagano, Japan; mio_kobayashi@shinshu-u.ac.jp; Tel.: +81-263-37-3509

**Keywords:** Rulkov map, neuronal network, Q-learning, discrete-time dynamical systems, signal transmission, chaos

## Abstract

The dynamics of signal transmission in neuronal networks remain incompletely understood. In this study, we propose a novel Rulkov neuronal network model that incorporates Q-learning, a reinforcement learning method, to establish efficient signal transmission pathways. Using a simulated neuronal network, we focused on a key parameter that modulates both the intrinsic dynamics of individual neurons and the input signals received from active neighbors. We investigated how variations in this parameter affect signal transmission efficiency by analyzing changes in attenuation rate, as well as the maximum and minimum firing intervals of the start and goal neurons. Our simulations revealed that signal transmission efficiency between distant neurons was significantly impaired in the parameter region, where a chaotic attractor and an attractor of the eight-periodic points are observed to co-exist. A key finding was that low-frequency oscillatory bursts, while failing long-distance transmission, were capable of amplifying signals in neighboring neurons. Furthermore, we observed variation in signal transmission even when individual neuron dynamics remained similar. This variability, despite similar presynaptic activity, is a biologically significant phenomenon, and it is argued that it may contribute to the flexibility and robustness of information processing. These findings are discussed in the context of their biological implications.

## 1. Introduction

Nonlinear phenomena observed in neurons have intrigued researchers for decades. Complex and unpredictable neuronal responses have been elucidated as deterministic phenomena that follow nonlinear dynamics principles [1,2,3,4]. Various proposed models have contributed to understanding and investigating these diverse dynamics [5,6,7,8,9].

Among these models, discrete-time dynamical systems are particularly interesting because they can effectively reproduce a wide range of complex phenomena, including spiking, bursting, and chaotic dynamics, similar to those generated by continuous-time ordinary differential equations (ODEs). Importantly, they require significantly less computational time for simulation and analysis. The Rulkov map [9,10,11] is one such discrete-time dynamical system. Described by a two-dimensional iterated map, it is utilized in diverse fields, not only in neuronal sciences but also in electronic engineering [12,13,14,15], economic dynamics [16], and the broader field of nonlinear dynamical systems [17,18,19]. While these models effectively capture intrinsic neuronal dynamics, understanding how such dynamical elements can form adaptive systems capable of learning and flexible information processing remains an important area of study.

To address this challenge, this study employs reinforcement learning (RL), particularly Q-learning [20]. Q-learning is a simple yet powerful model-free RL algorithm that enables an agent to learn optimal actions through iterative trial and error, without requiring explicit knowledge of environmental dynamics described by transition probabilities. In recent years, research integrating RL with spiking neural networks (SNNs) has become increasingly common. These studies often focus on task-specific applications such as robotic control, motion planning, and image recognition [21,22,23,24]. They typically treat the SNN as a computationally efficient substrate for an RL agent, and their primary goal is to achieve high performance on a given task. In contrast, our study investigates more fundamental perspectives by incorporating intrinsic dynamics. Specifically, we examine how nonlinear neurons autonomously self-organize for efficient signal transmission, and how an individual neuron’s dynamics affect signal transmission within a coupled network. This study aims to model the autonomous development of functional information transmission routes, such as optimal signal transmission paths, within a neuronal network. This is achieved by utilizing the Q-learning method on a Rulkov neuron network, where Q-learning guides each neuron to learn how to choose its neighboring neurons for signal transmission. The Rulkov map is adopted in this study due to its capability of generating diverse neuronal oscillatory responses by varying parameter values, and its computational efficiency as a discrete-time dynamical system. These advantages simplify the implementation of the Q-learning method into the neuronal network model.

The main focus of this study is to elucidate how individual neuronal dynamics affect information transmission within the neuronal network, where neurons autonomously learn to optimize signal transmission. In other words, the core of this study lies in the integration between individual neuronal dynamics and reinforcement learning at the network level.

The key contributions of this study are as follows:

- Proposes a novel computational model for neuronal networks, comprising Rulkov neurons exhibiting nonlinear dynamics, where Q-learning autonomously configures signal transmission pathways based on learned Q-values.
- Quantitative analysis of how intrinsic neuronal dynamics (e.g., bursting and spiking) affect signal transmission.

The remainder of this paper is organized as follows: Section 2 describes the mathematical formulation of the Rulkov neuron model and Q-learning; Section 3 details the simulation environment and the method for applying the Q-learning algorithm to the neuronal network model, as well as the signal transmission analysis method used in this study; Section 4 presents the simulation results and analyzes the learned signal transmission pathways and the dynamic behavior of neurons under various parameter conditions; Section 5 discusses the analysis results from Section 4; Section 6 concludes this study and discusses future prospects.

## 2. Preliminaries

This section details the Rulkov map and Q-learning framework utilized in this study.

### 2.1. Rulkov Map

**Figure 1:**
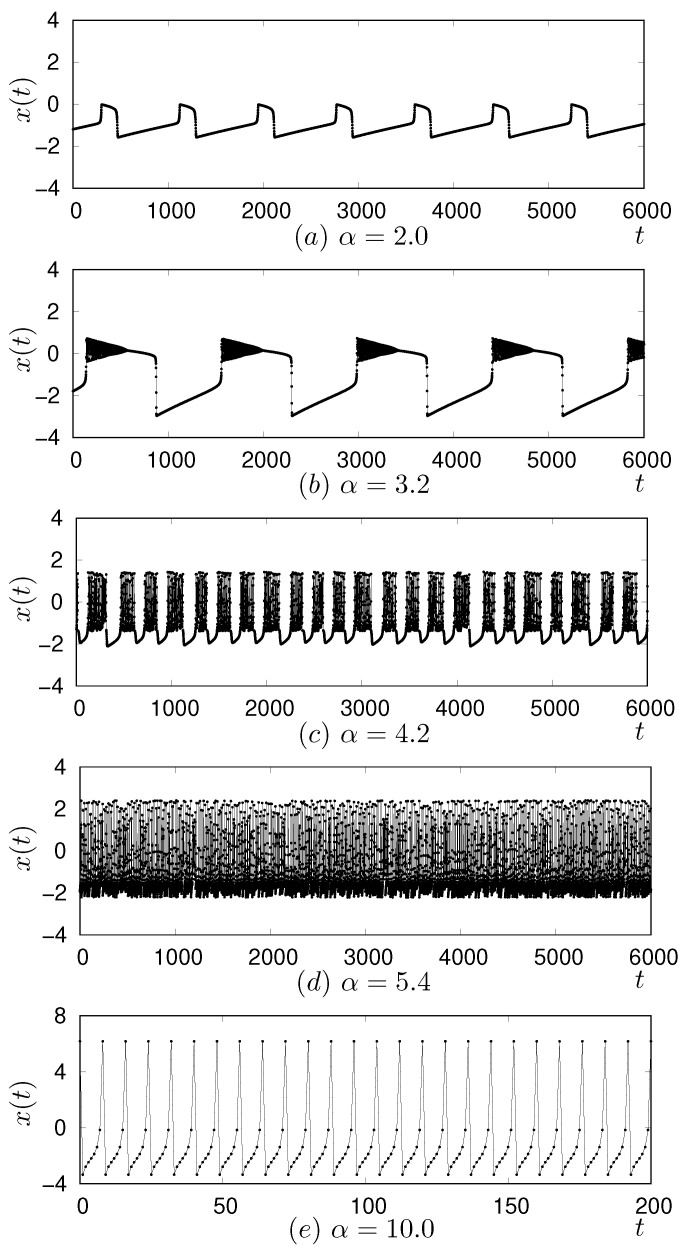
The behavior of the Rulkov map depending on the parameter α. Parameters μ, and σ are set to 0.001.

The Rulkov map is a two-dimensional iterated map that generates various neuronal oscillation patterns, described by the following equations:

(1) $$\begin{array}{ccc} x(t+1) & = & \frac{\alpha }{1+x{\left(t\right)}^{2}}+y\left(t\right),\\ y(t+1) & = & y(t)-\mu x(t)-\sigma , \end{array}$$

where α, μ, and σ are parameters. Here, x(t) represents the fast dynamics of the neuron’s membrane potential, while y(t) is a slow variable. This separation of timescales is particularly evident when the parameters μ and σ are set to very small values. In this study, we set both μ and σ to 0.001. Depending on the value of parameter α, the Rulkov map generates various types of oscillatory and chaotic behavior, as illustrated in Figure 1. For instance, Figure 1a–d show examples where α was set to 2.0, 3.2, 4.2, and 5.4, respectively, demonstrating transitions in dynamics.

**Figure 2:**
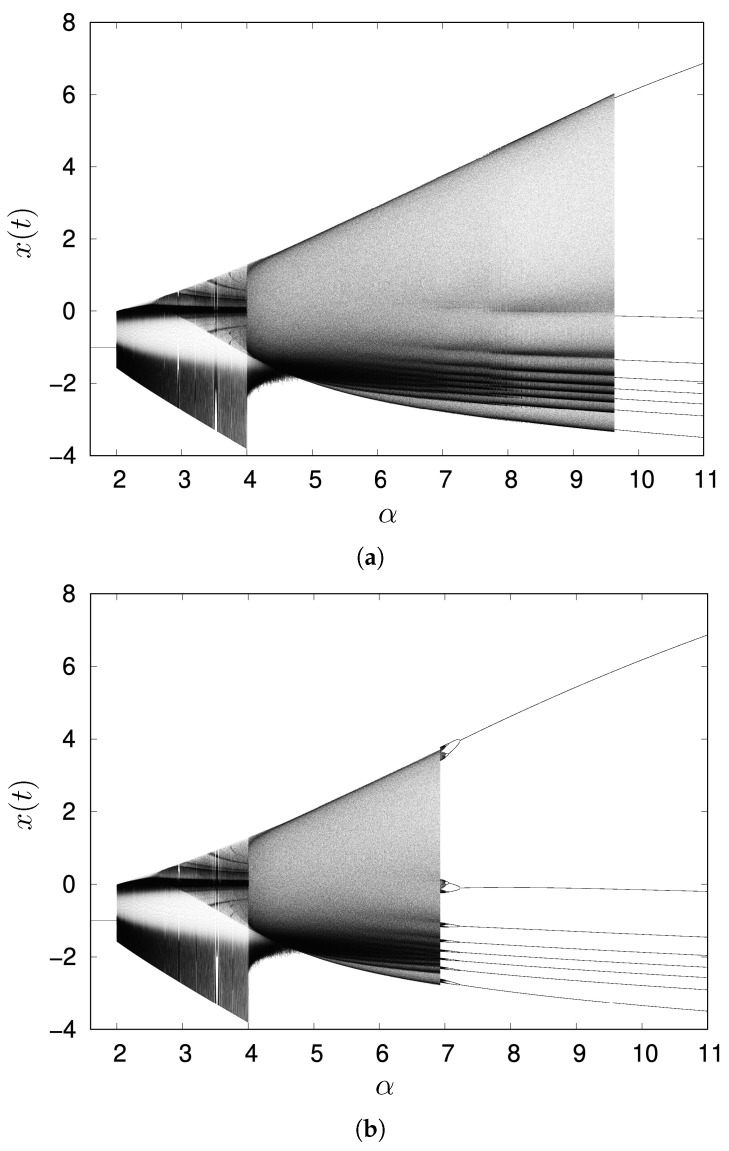
One parameter bifurcation diagrams with μ=σ=0.001. Initial values are set to (x(0),y(0))=(−0.415458,−2.814479). (**a**) The parameter α was varied in an increasing manner for the data, and (**b**) in a decreasing manner for the data.

Figure 2 presents one-dimensional bifurcation diagrams for the parameter α. The initial conditions for the first α value in each sweep were set to (x(0),y(0))=(−0.415458,−2.814479). This specific point was chosen to ensure the system is initialized in a state close to the chaotic attractor for α=4.2. For each α value, we performed 5000 iterations and then recorded the last 1000 data points to generate the bifurcation diagram. For subsequent α values, the final state (x(t),y(t)) at t=5000 from the preceding parameter value was used as the initial condition. Figure 2a shows data obtained by increasing α; Figure 2b shows data obtained by decreasing α.

When the parameter α is less than approximately 2.0, a stable fixed point emerges, indicating that the map dynamics do not exhibit firing. As α ranges from approximately 2.0 to below 4.0, the system dynamics initially exhibits relatively periodic and slow oscillations, as depicted in Figure 1a. As α increases from around 3.0 and approaches 4.0, these oscillations become superimposed with burst firing, as illustrated in Figure 1b. Beyond approximately α = 4.0, the system exhibits intermittent spiking-bursting firing, as shown in Figure 1c. As the parameter further increases, this intermittency diminishes, leading to a transition towards sustained bursting firing, as depicted in Figure 1d. In the parameter region, from approximately α=7.0 to 9.5, both chaotic behavior and an eight-periodic attractor can be observed, depending on the initial values of variables (x(0),y(0)), as shown in Figure 2. With a further increment of the parameter α, only an eight-periodic attractor was observed, as shown in Figure 1e.

### 2.2. Q-Learning

Q-learning is one of the most important algorithms in reinforcement learning, utilized across a wide range of fields such as robotics control, game AI, and optimization problems. The basic concept of Q-learning involves an agent learning Q-values, which represent the accumulated rewards expected after taking a specific action in a given state. By choosing actions that maximize the Q-value, the agent learns an appropriate strategy for interacting with its environment.

Here, we review the general idea of Q-learning, which comprises four main stages. The first stage begins with the initialization of a Q-table, where Q-values, serving as indicators of accumulated rewards expected for taking a specific action in a given state, are stored. In the second stage, an agent selects an action from multiple available options. In this situation, the agent has two choices:

- With a probability of (1−ε), it exploits its current knowledge by choosing the action corresponding to the highest Q-value for the current state.
- With a probability of ε, it explores by choosing a random action from all possible actions in that state.

This strategy is known as the ε-greedy method. At the beginning of the learning process, ε is generally set to a large value (e.g., 0.9 or 1.0) to encourage the agent to explore various options flexibly. Towards the end of the learning process, ε should be reduced to a small value, as this phase focuses on utilizing the learned Q-values to maximize rewards.

In the third stage, the agent applies the selected action $A_{t}$, which results in a transition from the current state $S_{t}$ to a new state $S_{t+1}$, and it receives a corresponding reward $R_{t+1}$. Lastly, in the fourth stage, the Q-value for the state-action pair $\left(S_{t},A_{t}\right)$ is updated. This update is based on the received reward $R_{t+1}$ and the maximum Q-value for any action $a^{\prime }$ in the new state $S_{t+1}$. The update is computed using the following equation:

(2) $$Q\left(S_{t},A_{t}\right)\leftarrow Q\left(S_{t},A_{t}\right)+\beta \left[R_{t+1}+\gamma \max_{a^{\prime }}Q\left(S_{t+1},a^{\prime }\right)-Q\left(S_{t},A_{t}\right)\right],$$

where $Q(S_{t},A_{t})$ represents the Q-value for taking action $A_{t}$ in state $S_{t}$, and $\max_{a^{\prime }}Q\left(S_{t+1},a^{\prime }\right)$ denotes the maximum Q-value achievable from any action $a^{\prime }$ in the state $S_{t+1}$. Here, β is the learning rate and γ is the discount factor. In this study, these parameters are fixed at β=0.1 and γ=0.9. These values were chosen to balance stable learning and long-term optimization. With a relatively small learning rate of β=0.1, the Q-values are updated gradually, which prevents instability and helps the algorithm converge. The discount factor, γ=0.9, is a value close to 1.0, which encourages the agent to prioritize long-term rewards over immediate ones.

## 3. Method

This section describes the simulation environment adopted in this study, the parameter settings utilized in the Rulkov network model, and the measurement of signal transmission in detail.

### 3.1. Simulation Setup and Parameters

**Figure 3:**
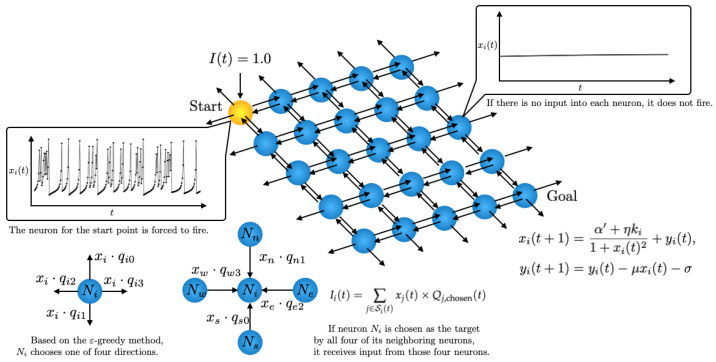
Schematic of the Q-learning model of a 5×5 Rulkov neuron network.

We implemented a network structure learned by Q-learning to solve a shortest path problem within a Rulkov neuron network. Through the Q-learning method, each neuron learns to determine the optimal outgoing connection for signal propagation and maintains its Q-values individually. It is important to note that while the output $x_{i}\left(t\right)$ of each Rulkov neuron serves as the signal for propagation, the intrinsic nonlinear dynamics of the Rulkov map do not directly influence the Q-learning algorithm’s update rule. A 5×5 grid of Rulkov neurons is utilized, where each neuron can connect to its neighboring neurons in the Von Neumann neighborhood. A schematic of the network is shown in Figure 3. The start and goal points are set at (0, 0) and (4, 4), respectively. In this Rulkov neuron network, the *i*th neuron is modeled by the following equations:

(3) $$\begin{array}{cc} x_{i}\left(t+1\right) & =\frac{\alpha^{\prime }+\eta k_{i}}{1+x_{i}{\left(t\right)}^{2}}+y_{i}\left(t\right),\\ y_{i}\left(t+1\right) & =y_{i}\left(t\right)-\mu x_{i}\left(t\right)-\sigma , \end{array}$$

where $k_{i}$ is a binary value defined as follows:

(4) $$k_{i}=\left\{\begin{array}{cc} 1 & \mathrm{if}\;I_{i}\left(t\right)\geq \theta ,\\ 0 & \mathrm{if}\;I_{i}\left(t\right)<\theta , \end{array}\right.$$

where *i* represents the index of each neuron $\left(i=1,2\ldots ,n^{2}\right)$. *t* indicates the discrete time step, and $I_{i}\left(t\right)$ is the total input value from neighboring neurons at time *t*. θ is a threshold set to 0.2. This specific value was selected based on preliminary simulations to ensure stable and observable firing patterns throughout the network. The parameter η in Equation (3) is an amplification factor of the input signal, which dynamically modulates the neuron’s firing patterns. The Rulkov neuron at the start point (0,0) receives an external input of $I_{1}\left(t\right)=1.0$ to initiate its firing at each time step. The input $I_{1}\left(t\right)=1.0$ was chosen as an arbitrary positive constant that is sufficiently large to reliably trigger the firing state. For neurons other than the one at the start point, the total input $I_{i}\left(t\right)$ is initialized to 0 at the beginning of each time step and then accumulates the signals received during that step, as shown in the following equation:

(5) $$I_{i}\left(t\right)=\sum_{j\in S_{i}\left(t\right)}x_{j}\left(t\right)\times Q_{j,\mathrm{chosen}}\left(t\right),$$

where $S_{i}\left(t\right)$ represents the set of all source neurons *j* that send a signal to neuron *i* at time *t*. $x_{j}\left(t\right)$ is the membrane potential of the source neuron *j*, and $Q_{j,\mathrm{chosen}}\left(t\right)$ is the Q-value chosen by source neuron *j* at time *t*. After $I_{i}\left(t\right)$ is used for the neuron’s update at time *t*, it is reset to 0 for the next time step. In Equation (3), we set the parameter values of each neuron as $\alpha^{\prime }=1.8$, μ=0.001, and σ=0.001. We chose the value of $\alpha^{\prime }=1.8$ to ensure the neurons remain at a fixed point instead of oscillating when they have no input from neighboring neurons. Furthermore, the values for μ and σ were chosen to be identical to those used in the original paper by Rulkov [10]. The signal transmission features are distinct, depending on the value of η, a point that will be discussed in Section 5.

In our model, instead of using a global Q-table, each Rulkov neuron maintains four Q-values, each corresponding to one of the four cardinal directions (up, down, left, right). These Q-values are initialized with random values in the range of 0.01 to 0.2. This initialization strategy was chosen to encourage diverse exploratory behaviors during the initial learning phase while avoiding significant initial bias in the Q-values. Starting with a small positive random value encourages the agent to explore a variety of paths without being overly influenced by a single high-value path. Each Rulkov neuron behaves as an individual agent and, based on its Q-values, determines which neighboring neuron to connect to. Specifically, this determination prioritizes the direction corresponding to the highest Q-value.

In the learning process, Rulkov neurons autonomously learn the shortest path for neuronal signal propagation using Q-learning. The Rulkov neuron network updates the Q-values maintained by each neuron through an iterative simulation of the 2000 episodes. The ε-greedy method is applied, where the ε value is updated after the *m*th episode by the following equation:

(6) $$\begin{array}{c} \varepsilon_{m}=\varepsilon_{\mathrm{ini}}\times p^{m}, \end{array}$$

where $\varepsilon_{\mathrm{ini}}$ represents the initial value of ε, and *p* is its decay rate. In the simulation, these values are set to $\varepsilon_{\mathrm{ini}}=1.0$ and p=0.999. When 2000 episodes are executed, the final value of ε is approximately 0.135. For the ε-greedy method, the initial setting of $\varepsilon_{\mathrm{ini}}=1.0$ ensures that the agent explores with a 100% probability during the first episode. The decay rate p=0.999 was chosen to ensure a gradual decrease in ε over the course of 2000 episodes, allowing for a smooth transition from a focus on exploration to a greater emphasis on exploiting learned knowledge. The final value of ε≈0.135 indicates that the agent retains a degree of exploratory behavior to avoid getting trapped in local optima even at the end of the simulation. The reward values *R*, used in Equation (2), are defined as follows:

- $R_{\mathrm{goal}}=10.0$: reward for reaching the goal state;
- $R_{\mathrm{action}}=-0.05$: cost per action;
- $R_{\mathrm{out}}=-1.0$: cost of moving out of bounds.

These reward values are constructed by combining a sparse reward and a dense reward. The large value of $R_{\mathrm{goal}}=10.0$, as the sparse reward, encourages the agent to learn that the significant objective is to achieve the goal. The dense rewards, $R_{\mathrm{action}}=-0.05$ and $R_{\mathrm{out}}=-1.0$, are designed to avoid unfavorable actions such as taking unnecessary steps or moving out of bounds. The cost for moving out of bounds is set to a larger negative value to strongly penalize invalid actions. At the beginning of each episode, $x_{i}\left(0\right)$ and $y_{i}\left(0\right)$ are initialized with random values within the ranges of (−0.415458±0.01) and (−2.814479±0.01), respectively. This randomization in initial conditions was introduced to demonstrate that the learning process is appropriate for not only a specific initial state, but also for a variety of initial conditions. However, comparative simulations without randomization for initial conditions showed no significant difference in the final results. This is because the random initialization of the Q-values already ensures the robustness of the learned policy regardless of the initial state of the Rulkov neurons.

### 3.2. Signal Transmission Measurement and Analysis

The oscillatory behavior of the start neuron and the signal transmission from the start to the goal vary depending on the parameter value of η as defined in Equation (3). To analyze this phenomenon, the attenuation rate of each neuron on the signal transmission path is calculated using the following equation:

(7) $$\begin{array}{ccc} \mathrm{Attenuation}\;\mathrm{Rate} & = & 10\log_{10}\left(\frac{A_{1}}{A_{i}}\right), \end{array}$$

where $A_{i}$ represents the sum of signal amplitudes greater than the firing threshold of 0.2 for the *i*th neuron on the path over 5000 steps, and $A_{1}$ represents the corresponding sum for the start neuron. If a neuron exhibits no signal greater than 0.2 over 5000 steps, $A_{i}$ becomes zero, which leads to an undefined (infinite) attenuation rate.

## 4. Results

**Figure 4:**
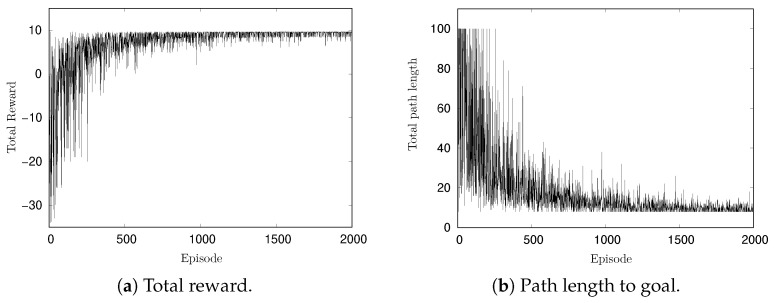
Change of rewards and path length per episode in simulation.

Figure 4a,b show the changes in total reward per episode and the path length to the goal per episode, respectively. In the early part of the simulation, the reward is approximately −30 and gradually increases with learning progress. The maximum reward observed was 9.6, which is consistent with the theoretical maximum for the optimal path. Given a reward of 10 for reaching the goal and a cost of −0.05 per action, the shortest path length of 8 in a 5×5 grid yields a maximum reward of 9.6 (=10+8×(−0.05)). These results confirm the successful execution of the learning process. In Figure 4b, at the beginning of the simulation, the path length from the start to the goal reached is 100 steps in some episodes. This is because the maximum path length per episode was set to 100. As the episode progresses, the path length converges to 8.

**Figure 5:**
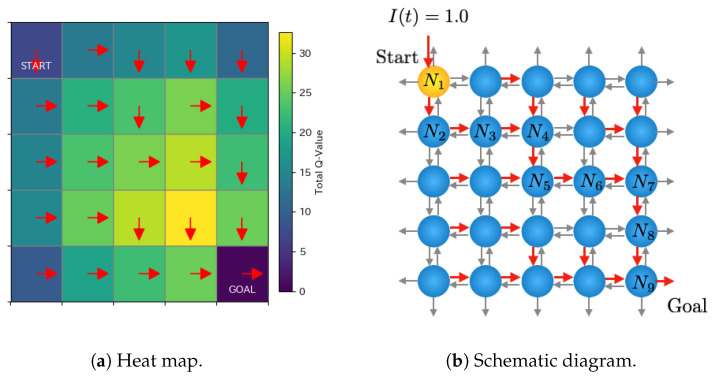
Typical example of learned Q-values after 2000 episodes. The red arrows indicate the direction corresponding to the highest Q-value.

Figure 5 shows an example of learned Q-values after 2000 episodes. Figure 5a represents a heat map corresponding to the total Q-values of each Rulkov neuron, and the red arrows indicate the direction corresponding to the highest Q-value. Figure 5b is the corresponding schematic diagram. The neurons labeled as $N_{i}$ indicate those on the path where the signal is transmitted from the start to the goal. For instance, the Q-values of the Rulkov neuron at the start point are [−1.05, 4.58, −1.05, 4.58], and those of the neuron at (3,3) are [7.24, 9.05, 7.24, 9.05]. These values are rounded to two decimal places for presentation, although they are calculated with full floating-point precision in the simulation. If multiple directions exhibit the same highest Q-values, one of them is chosen randomly as the direction for signal transfer.

**Figure 6:**
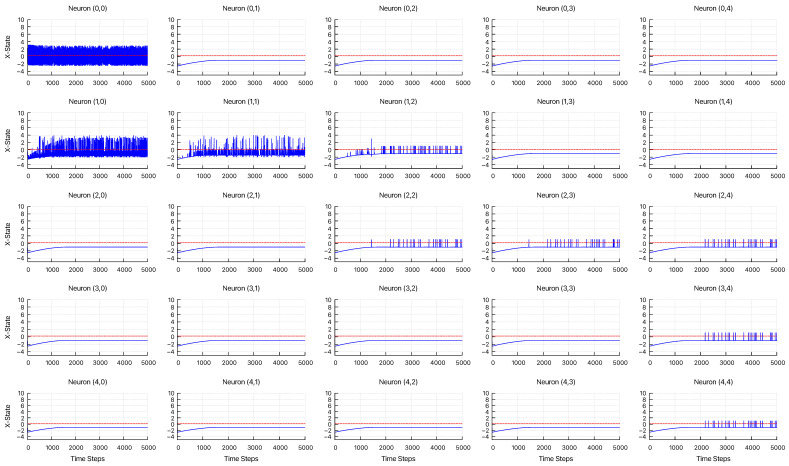
Time-series dynamics of *X*-state for 25 neurons, showing their response to the input, with $\alpha^{\prime }=1.8$, η=4.2, μ=0.001, and σ=0.001. The red dashed lines indicate the firing threshold, θ=0.2.

Figure 6 displays the time series dynamics of $x_{i}\left(t\right)$, as defined in Equation (3). The network topology corresponding to this simulation is illustrated in Figure 5. The parameters were set to $\alpha^{\prime }=1.8$, η=4.2, μ=0.001, and σ=0.001. For neurons that do not fire, their state variables converge to the fixed point at (x(t),y(t))=(−1.0,−1.9). Additionally, the start neuron receives an external input at each time step. The red dashed line indicates the firing threshold of $x_{i}=0.2$, which was used for each neuron in the attenuation analysis. The results indicate that signal decay occurs depending on the distance from the start neuron.

**Figure 7:**
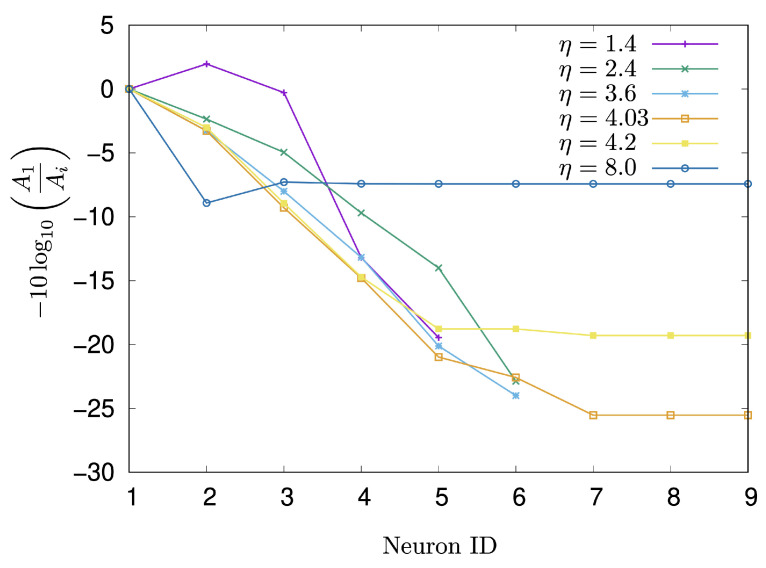
Change in the attenuation rate of each neuron on the signal transmission path over 5000 iterations at $\alpha^{\prime }=1.8$.

To elucidate the mechanism of signal decay, the attenuation rate, as defined in Equation (7), was investigated with respect to the parameter η. Figure 7 displays the changes in the attenuation rate for each neuron along the signal transmission path from the start to the goal. The parameters were set as $\alpha^{\prime }=1.8$, μ=0.001, and σ=0.001. The neuron IDs in Figure 7 correspond to the $N_{i}$ labels in Figure 5b. When η was set to 1.4,2.4, and 3.6, the signal could not reach the goal neuron. Interestingly, however, at η=1.4, the signal observed at $N_{2}$ was amplified compared to that at $N_{1}$. At η=4.03, the signal decays gradually from $N_{1}$ to $N_{7}$, after which the attenuation rate becomes constant. The situation at η=4.2 is similar to that at η=4.03, but the constant value of the attenuation rate is lower than that observed at η=4.03. When η=8.0, the attenuation rate reaches its peak of approximately 8.9 at $N_{2}$, and subsequently converges around 7.4.

**Figure 8:**
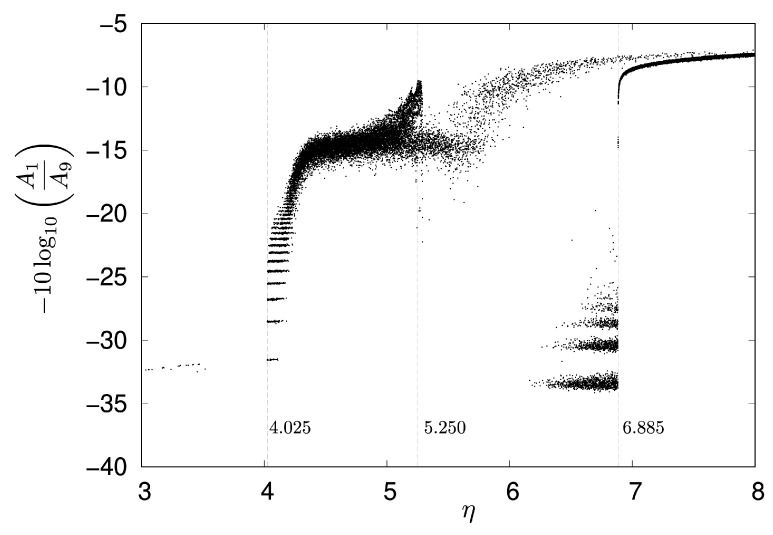
Attenuation rate of $N_{9}$ with respect to η, with $\alpha^{\prime }=1.8$, μ=0.001, and σ=0.001.

To investigate the attenuation rate at the goal neuron $N_{9}$ in detail, for each value of η, a signal transmission simulation was conducted 10 times, with each run lasting 5000 steps. The parameter η was varied from 3.00 to 8.00 with an interval of 0.001. Figure 8 shows the change in the attenuation rate of the goal neuron $N_{9}$, with dashed lines indicating η=4.025, 5.250, and 6.885, respectively. When the parameter η is approximately 4.024 or less, the input signal from neuron $N_{1}$ rarely reaches $N_{9}$. Specifically, the signal transmission rate varied significantly across different ranges of η, as shown in Figure 8:

- For η in the range of 3.000 to 4.024, the transmission rate was 0.20%.
- A dramatic increase in transmission rate was observed for η between 4.025 and 5.249, reaching 99.9%.
- In the range of 5.250 to 6.884, the transmission rate decreased to 23.8%.
- Finally, for η from 6.885 to 8.000, the transmission rate achieved 100%.

Given that the waveform observed in neuron $N_{1}$ is equivalent to that of a single Rulkov neuron described by Equation (3) with a parameter $\alpha =\alpha^{\prime }+\eta =1.8+\eta$, it follows that η=4.025,5.250, and 6.885 correspond to α=5.825,7.050, and 8.685, respectively.

**Figure 9:**
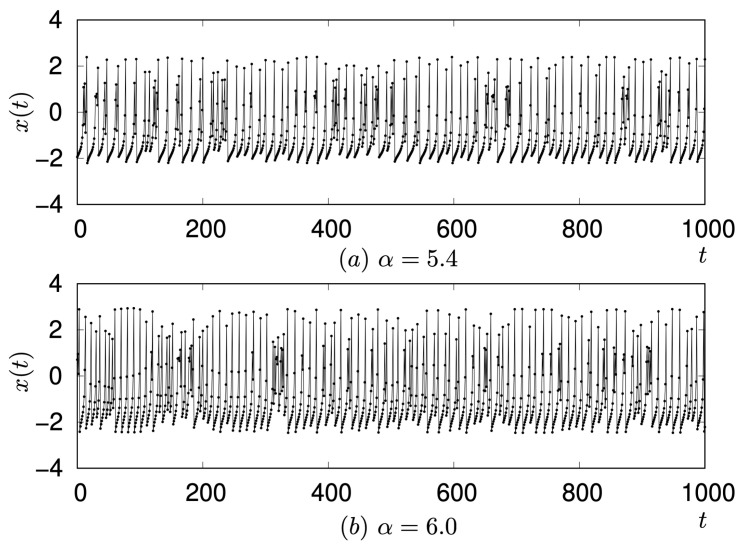
Comparison of waveforms of a single Rulkov neuron observed at α=5.4 and α=6.0.

As shown Figure 7, a notable difference in signal transmission was observed when comparing η=3.6 and η=4.2: neuron $N_{9}$ fired at η=4.2 but did not fire at η=3.6. For these η values, the waveform of neuron $N_{1}$ corresponds to a single Rulkov neuron with $\alpha =\alpha^{\prime }+\eta =1.8+3.6=5.4$ and 1.8+4.2=6.0, respectively. Although the intrinsic dynamics of a single Rulkov map at α=5.4 and α=6.0 are largely similar, differing mainly in amplitude (as represented in Figure 9), their consequences for signal transmission within the network are strikingly different.

**Figure 10:**
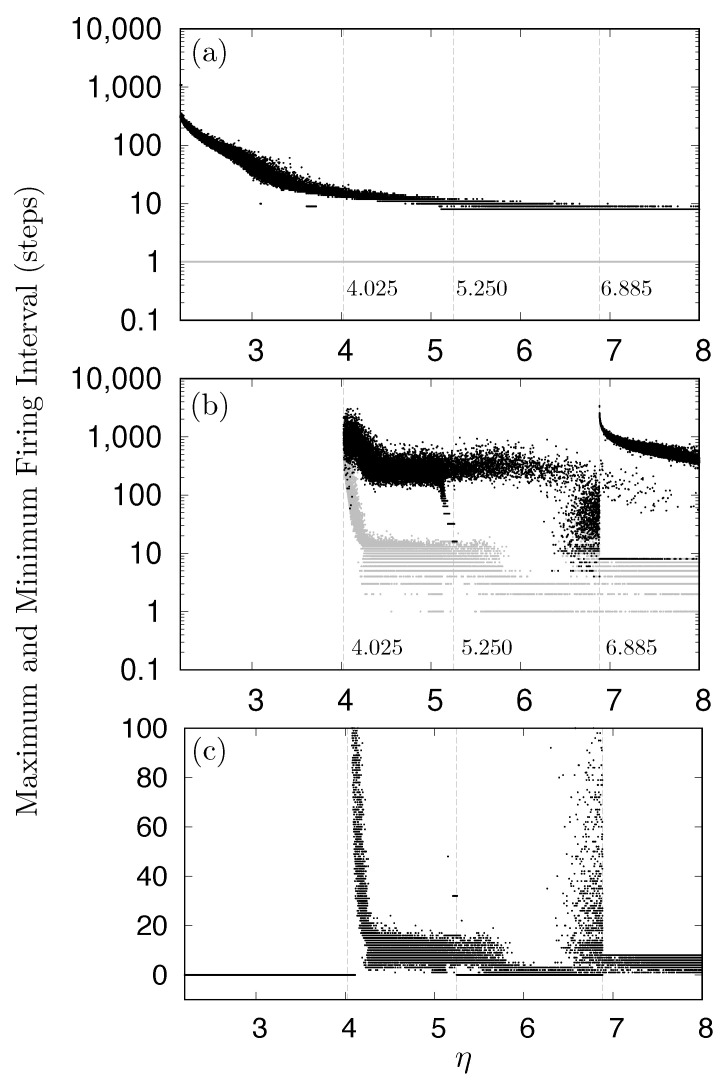
Relationship between η and firing intervals. (**a**) Maximum and minimum firing intervals for neuron $N_{1}$. (**b**) Maximum and minimum firing intervals for neuron $N_{9}$. (**c**) Minimum firing intervals for neuron $N_{9}$, where zero values indicate periods when the neuron did not fire. In panels (**a**,**b**), gray dots represent minimum firing intervals, while black dots represent maximum firing intervals.

To further analyze these phenomena, the relationship between η and the firing intervals for neuron $N_{1}$ and $N_{9}$ was investigated, as shown in Figure 10. A neuron was considered to be firing when its activity level (x-state) exceeded 0.2. Under the same measurement conditions as for Figure 8, both the maximum and minimum firing intervals were recorded. Figure 10a,b illustrate the maximum and minimum firing intervals, for neurons $N_{1}$ and $N_{9}$, respectively, shown by black and gray dots with a logarithmic vertical scale. Figure 10c depicts the minimum firing intervals for neuron $N_{9}$ with a linear vertical scale. For the start neuron $N_{1}$, as shown in Figure 10a, the minimum firing interval constantly remained at one, while the maximum intervals varied widely from eight to approximately 500. As shown in Figure 10b,c, neuron $N_{9}$ did not fire during any of the 10 simulation runs when η was approximately 4.024 or less, as indicated by the zero values in Figure 10c. Furthermore, Figure 10b,c demonstrate that the dynamics of neuron $N_{9}$ drastically changed around η=4.025 and η=6.885.

## 5. Discussion

This study implemented a Rulkov neuronal network where Q-learning was used to learn the shortest signal transmission path. Simulations were then conducted by varying the parameter η, which acts as an amplification factor and a dynamical modulation parameter for the input signal received by a neuron when its neighbors fire. The findings suggest that the intrinsic nonlinear dynamics of individual neurons play a significant role in network-level information processing. In this section, we discuss the findings in relation to neuron dynamics. Furthermore, strengths, limitations, and future work are summarized.

### 5.1. Interpretation of Findings in Relation to Neuron Dynamics

Simulations revealed a direct link between the network’s signal transmission efficiency and the dynamical properties of the individual Rulkov neurons. In the simulations, to investigate the effect of the amplification factor η on signal transmission, the attenuation rate, as defined in Equation (7), was calculated across different η values. For instance, as illustrated in Figure 8, when η is set to less than 4.025, the input signal from $N_{1}$ rarely reaches the goal neuron $N_{9}$. Through the simulation of the signal transmission, $\alpha^{\prime }$ was set to 1.8 to ensure the neurons remain at a fixed point instead of oscillating when they have no input from neighboring neurons.

Within the parameter region of η=5.250 to 6.884, which corresponds to an α range of 7.050–8.685 for a single Rulkov neuron, the observed low transmission rate is likely attributed to the cascade of period-doubling bifurcations originating around α=7.050 (as shown in Figure 2b). In this parameter region, a chaotic attractor and an attractor of the eight-periodic points are observed to co-exist. Low-frequency oscillatory bursts from a neuron failed to transmit to a goal neuron eight paths away, yet they amplified the signal of a connected neighboring neuron. This observation aligns with the concept in [25], where bursts are considered a unit of neural information that makes unreliable synapses reliable by enhancing neurotransmitter release.

Furthermore, as discussed in [26], neuronal mechanisms, particularly in thalamic relay cells, can dynamically switch between tonic (single spike) and burst firing modes. This suggests that not only single spike firings are filtered, but also that specific firing patterns (e.g., bursts or tonic spikes) are selectively transmitted or filtered based on functional requirements.

Notably, our simulations revealed that seemingly similar presynaptic firing patterns can lead to variable signal transmission to target neurons. This variability, despite similar presynaptic activity, is a biologically significant phenomenon, and it is argued that it may contribute to the flexibility and robustness of information processing [27,28]. While our simulation results clearly demonstrate that an input signal from $N_{1}$ rarely reaches the goal neuron $N_{9}$ when η is set to less than 4.025 in our model, the precise underlying mechanism for this behavior remains to be fully elucidated within the scope of the current study, requiring further investigation.

### 5.2. Strengths, Limitations, and Future Work

The Rulkov model offers a distinct computational advantage over other prominent neuron models, such as the Hodgkin–Huxley [1], Leaky Integrate-and-Fire (LIF) [29], and Izhikevich models [30]. While the Hodgkin–Huxley model provides high biological realism, its significant computational cost makes it impractical for large-scale network simulations. On the other hand, the LIF model is computationally efficient but fails to reproduce rich and varied firing patterns, such as bursting dynamics that are characteristic of many biological neurons. The Izhikevich model strikes a good balance between biological realism and computational efficiency. However, it is a continuous-time model, meaning its values must be obtained through numerical integration (for example, using the Euler method). This process can introduce numerical integration errors that may compromise the accuracy of the simulation results. From this perspective, the Rulkov model is a suitable choice. As a discrete-time dynamical system, it inherently avoids the numerical integration errors that affect continuous-time models. Furthermore, it is exceptionally computationally efficient and can reproduce a wide variety of complex firing phenomena with a simple mathematical formulation.

The integration of the Q-learning algorithm with the Rulkov neuronal network provides an analogy to biological learning. In the model, the Q-value for each synaptic connection is updated based on a reward that strengthens optimal pathways. This process analogizes to Hebb’s rule [31,32], which is often summarized as “neurons that fire together, wire together,” where synaptic strength is modified by the correlated firing of pre- and post-synaptic neurons. Despite this analogy to Hebb’s rule, our current model does not yet fully replicate the biological phenomenon of synaptic strengthening based on the synchronous firing of neurons. Our approach simplified the problem by modeling connections for a shortest-path problem with a defined start and end neuron. Therefore, key future challenges are to design a reinforcement learning framework that is based on the synchronization of the firing phenomena of the neurons themselves. This would involve proposing, for instance, a model where learning enables neurons on a specific pathway to burst synchronously to cooperatively transmit important information.

Crucially, in the proposed model, the parameter *k* acts as a dynamic gatekeeper for signal propagation. When a neuron receives a sufficient input signal from neighboring neurons, *k* turns on, conceptually ‘opening the gate’ for signal flow. This is dependent on the established coupling learned by the Q-learning algorithm. This can be seen as an analogy to the gating mechanisms of ion channels in biological neurons [33,34], and it can be interpreted as the network making a decision to activate a specific connection to transmit a particular piece of information, and encoding that decision.

However, we acknowledge that our approach has limitations. Our current model does not account for the complexities of real-world biological systems, such as the full spectrum of synaptic plasticity [35,36], which is a key mechanism for learning and memory. Furthermore, while the Rulkov model effectively reproduces a variety of firing patterns, it is a phenomenological model and does not fully capture the detailed biophysical mechanisms of ion channels. Therefore, our findings should be interpreted as a proof-of-concept for how a simple learning framework can be applied to nonlinear neuronal dynamics.

## 6. Conclusions

In this study, we proposed a novel computational model for neuronal networks that combines Rulkov neurons with the Q-learning algorithm. This model enabled the development of connections among neurons, allowing each neuron to learn optimal outgoing connections to its neighbors based on its learned Q-values. With the established shortest path, a qualitative analysis was conducted to investigate how the input signal from neuron $N_{1}$ was transmitted to the goal neuron $N_{9}$, and how intrinsic neuronal dynamics affected this signal transmission. Our analysis revealed a direct and significant link between the intrinsic nonlinear dynamics of individual neurons and network-level signal transmission efficiency. Specifically, our simulations demonstrated that when parameter η is less than 4.025, the input signal from the start neuron $N_{1}$ does not reach the goal neuron $N_{9}$ in the model. This is because bursts with low-frequency oscillations, while effective for transmitting signals to immediate neighbors, may not be robust enough to propagate to distant neurons. To fully elucidate the underlying mechanism of this phenomenon, further investigation into the basin of attraction of each attractor is necessary, involving observation of the dynamics of not only neurons at the start and goal points but also other neurons along the signal transmission path. Future work will focus on exploring learning frameworks that are based on the synchronous firing of neurons and further investigating the role of dynamic gating mechanisms. This research provides a crucial foundation for understanding how the intrinsic dynamics of individual neurons can be leveraged to optimize signal transmission in larger networks. 

## Data Availability

Data are contained within the article.
